# Weyl’s Law for the Steklov Problem on Surfaces with Rough Boundary

**DOI:** 10.1007/s00205-023-01912-6

**Published:** 2023-08-10

**Authors:** Mikhail Karpukhin, Jean Lagacé, Iosif Polterovich

**Affiliations:** 1 Department of Mathematics, University College London, Gower Street, London, WC1E 6BT UK; 2 Department of Mathematics, King’s College London, The Strand, London, WC2R 2LS UK; 3 Département de Mathématiques et de Statistique, Université de Montréal, CP 6128, succ. Centre-ville, Montréal, QC H3C 3J7 Canada

## Abstract

The validity of Weyl’s law for the Steklov problem on domains with Lipschitz boundary is a well-known open question in spectral geometry. We answer this question in two dimensions and show that Weyl’s law holds for an even larger class of surfaces with rough boundaries. This class includes domains with interior cusps as well as “slow” exterior cusps. Moreover, the condition on the speed of exterior cusps cannot be improved, which makes our result, in a sense optimal. The proof is based on the methods of Suslina and Agranovich combined with some observations about the boundary behaviour of conformal mappings.

## Introduction and Main Results

### Asymptotics of Steklov Eigenvalues

Let $\Omega$ be a bounded domain in a smooth complete Riemannian manifold $(\mathcal {M},g)$ of dimension $d \geqslant 2$. Consider the Steklov eigenvalue problem

1.1 $$\begin{aligned} {\left\{ \begin{array}{ll} \Delta u = 0 &{} \text {in } \Omega ; \\ \partial _\nu u = \sigma u &{} \text {on } \partial \Omega , \end{array}\right. } \end{aligned}$$

where $\Delta$ is the Laplace-Beltrami operator on $\mathcal {M}$ associated with the Riemannian metric *g* and $\partial _\nu$ is the outward normal derivative. Under some regularity conditions on the boundary, for instance $\partial \Omega$ Lipschitz [3], the spectrum is discrete and forms a sequence accumulating only at infinity:

$$\begin{aligned} 0 = \sigma _0(\Omega ) \leqslant \sigma _1(\Omega ) \leqslant \sigma _2(\Omega ) \leqslant \cdots \nearrow \infty . \end{aligned}$$

We will discuss weaker conditions under which the spectrum is discrete later on. To study eigenvalue asymptotics it is convenient to introduce the eigenvalue counting function

$$\begin{aligned} N(\sigma ) := \# \left\{ j \in {\mathbb {N}}: \sigma _j(\Omega ) < \sigma \right\} . \end{aligned}$$

If $\partial \Omega$ is piecewise $\textrm{C}^1$, it is known [1] that the counting function satisfies the Weyl asymptotics

1.2 $$\begin{aligned} N(\sigma ) = \frac{\omega _{d-1}}{(2\pi )^{d-1}} \text {Vol}_{d-1}(\partial \Omega )\sigma ^{d-1} + o_{}\left( \sigma ^{d-1} \right) , \end{aligned}$$

where $\omega _d$ is the volume of the *d*-dimensional unit ball. We refer also to [2, 36, 37, 42] for earlier results on this topic, as well as to [11, 14, 15, 33] for improvements of the error estimate under stronger regularity assumptions. Extending the asymptotic formula (1.2) to domains with Lipschitz boundaries is a well known open problem, see for example [14, 17, 35, 43], to which we provide an answer in two dimensions.

#### Theorem 1.1

Let $\Omega$ be a bounded domain with Lipschitz boundary in a smooth complete Riemannian manifold of dimension two. Then its Steklov eigenvalues satisfy the asymptotics (1.2).

In fact, we prove that (1.2) holds for domains satisfying weaker regularity conditions defined via the boundary behaviour of conformal maps, see Sect. 1.2. We give examples of domains satisfying those conditions in Sect. 4, they include the so-called chord-arc domains, as well as domains with inward and “slow” outward cusps, see Proposition 4.2.

The proof of Theorem 1.1 relies on the variational characterisation of Steklov eigenvalues. While this characterisation is standard for Lipschitz domains, certain subtleties arise for domains with less regular boundary which we clarify in Sect. 1.3. We use a conformal map to obtain an isospectral weighted Steklov problem on a surface with smooth boundary. The isospectrality follows from the equivalence of the corresponding variational characterisations; for Lipschitz domains, it can be deduced almost directly from the analogous results for the Neumann problem [19]. Finally, we use the methods developed in [9, 42], see also [1], in order to obtain spectral asymptotics for these weighted Steklov problems. For Lipschitz domains we could use the results from [1, 42] in a straightforward way; we extend these techniques to allow for more singular weights corresponding to less regular boundaries.

#### Remark 1.2

While the present paper was under review, Theorem 1.1 was extended to higher dimensions in [34]. In this preprint, the variational ideas from [9] are also used after a reduction to a model problem. Since the theory of conformal maps cannot be applied in higher dimensions, it is replaced with a delicate and technically sophisticated analysis of elliptic operators with discontinuous coefficients.

### Conformal Regularity

As was mentioned above, our first goal is to reduce the Steklov problem on a surface with rough boundary to a weighted Steklov problem on a surface with smooth boundary, via a conformal map. Slightly abusing terminology, we refer to domains in two-dimensional Riemannian manifolds whose boundary is a finite collection of disjoint closed simple curves as *surfaces with boundary*. We say that two surfaces $\Omega _1$ and $\Omega _2$ with (potentially empty) boundary are *conformally equivalent*, or *in the same conformal class* if there exists $\varphi : \Omega _1 \rightarrow \Omega _2$ a conformal diffeomorphism of their interior extending to a homeomorphism of their boundary. This defines an equivalence relation on surfaces with boundary, and it is clear that every conformal class consists of surfaces with the same topological type, that is same orientability, genus and number of boundary components.

The uniformisation theorems are concerned with finding a canonical representative in every conformal class $\mathcal {C}$. These canonical representatives are *circle domains*, which are the complement of *b* geodesic disks in a closed surface $\mathcal {M}_\mathcal {C}$ endowed with a metric of constant curvature $g_\mathcal {C}$. It follows from the uniformisation theorems of Haas and Maskit [20, 25] that there is a circle domain in every orientable conformal class with finite topology; this result was extended to the non-orientable setting in [24, pp. 11–12]. We shall denote this canonical representative $(\Omega _\mathcal {C},g_\mathcal {C})$.

Many boundary regularity results in the litterature are proven for conformal maps from the disk to simply connected surfaces with boundary. It follows from [5, p.24] that any such result for maps from the disk is also valid for maps from an annulus into a doubly connected surface with boundary, by alternately filling the boundary components of the target with a disk and conjugating with inversions. As observed in [24, Remark 2.2], this allows one to extend the regularity theory to conformal maps from arbitrary circle domains with finite topology. Indeed, in that situation the restriction of the conformal map to a neighbourhood of one boundary component in the circle domain is a map from an annulus into a doubly connected surface with boundary. In particular, Carathéodory’s Theorem tells us that any conformal diffeomorphism of the interiors $\varphi : \Omega _\mathcal {C}\rightarrow \Omega$ extends to a homeomorphism of the boundary [30, Theorem 2.6].

#### Definition 1.3

Let $\mathcal {C}$ be a conformal class and $\varphi : \Omega _\mathcal {C}\rightarrow \Omega$ be a conformal diffeomorphism. We define, when they exist,

$$\begin{aligned} \beta := \left|\textrm{d}\varphi \big |_{\partial \Omega _\mathcal {C}} \right| \qquad \text {and} \qquad \eta := \left|\textrm{d}\varphi \right|^2. \end{aligned}$$

We call $\beta$ the *boundary conformal factor* and $\eta$ the *interior conformal factor*.

The interior conformal factor $\eta \in \textrm{L}^1(\Omega _\mathcal {C})$ and the Riemannian volume measure $\textrm{d}v_g$ on $\Omega$ is the pushforward measure $\varphi _*(\eta \, \textrm{d}v_{g_\mathcal {C}})$. If a surface with boundary has finite perimeter the boundary conformal factor $\beta \in \textrm{L}^1(\partial \Omega _\mathcal {C})$, and the boundary length measure $\textrm{d}\ell _g$ on $\partial \Omega$ is the pushforward measure $\varphi _*(\beta \, \textrm{d}\ell _{g_\mathcal {C}})$ [30, Theorem 6.8]. Integrability properties of the conformal factors $\beta$ and $\eta$ are controlled by the regularity of the boundary $\partial \Omega$. This motivates the following definition of regularity classes:

#### Definition 1.4

Let $\mathcal {C}$ be a conformal class, $\Omega \in \mathcal {C}$, and $\mathcal {X}(\Omega _\mathcal {C})$ and $\mathcal {Y}(\partial \Omega _\mathcal {C})$ be function spaces. We say that $\Omega$ has *boundary conformal regularity* $\mathcal {Y}$ if for some conformal diffeomorphism $\varphi : \Omega _\mathcal {C}\rightarrow \Omega$, the boundary conformal factor $\beta \in \mathcal {Y}$. We say that $\Omega$ has *interior conformal regularity* $\mathcal {X}$ if for some conformal diffeomorphism the interior conformal factor $\eta \in \mathcal {X}$.

We note that our definition of interior conformal regularity differs from that in [18, 19] by a factor of 2 since in those papers it was stated in terms of the integrability of $\left|\textrm{d}\varphi \right|$ rather than $\left|\textrm{d}\varphi \right|^2$. In other words, interior conformal regularity $\textrm{L}^p$ corresponds to domains which are $\textrm{L}^{2p}$ conformally regular in their definitions.

The integrability class of the interior conformal factor $\Omega$ has been used to investigate the properties of the Neumann Laplacian, see [18, 19]. As expected, the regularity of the boundary conformal factor also appears in the study of the Steklov problem.

#### Remark 1.5

By the Kellogg–Warschawski Theorem [30, Theorem 3.6], surfaces with boundary of class $\textrm{C}^{n,\alpha }$, $n \geqslant 1$ and $0< \alpha < 1$, have boundary conformal regularity $\textrm{C}^{n-1,\alpha }$. It follows furthermore from the arguments in the proof of [4, Lemma 5.1] that any surface with Lipschitz boundary (or, more generally, a chord–arc domain, see Sect. 4) has boundary conformal regularity $\textrm{L}^p$ for some $p > 1$, and surfaces of finite perimeter have boundary conformal regularity $\textrm{L}^1$.

Note that domains with exterior cusps do not have boundary conformal regularity $\textrm{L}^p$ for any $p>1$. In order to include some of these domains in our analysis we need to recall the following definition [6, Sections IV.6, IV.8]:

#### Definition 1.6

Given $(\Xi ,\mu )$ a measure space of finite measure and $a \geqslant 0$ the space $\textrm{L}(\log \textrm{L})^a (\Xi )$ is a space of functions *f* on $\Xi$ such that

$$\begin{aligned} \int _\Xi |f| (\log (2+|f|)^a \, \textrm{d}\mu < \infty \end{aligned}$$

endowed with the norm

$$\begin{aligned} \Vert f\Vert _{\textrm{L}(\log \textrm{L})^a (\Xi )}=\inf \left\{ t>0: \int _\Xi \left|\frac{f}{t} \right| \left( \log \left( 2+\left| \frac{f}{t}\right| \right) \right) ^a \, \textrm{d}\mu \leqslant 1\right\} . \end{aligned}$$

One can show that $\textrm{L}(\log \textrm{L})^a(\Xi )$ is a Banach space for every $a \geqslant 0$. The dual of $\textrm{L}(\log \textrm{L})^a(\Xi )$ is the space $\exp \textrm{L}^{1/a}(\Xi )$ of functions *f* on $\Xi$ such that

1.3 $$\begin{aligned} \left\| f \right\| _{\exp \textrm{L}^{1/a}(\Xi )} := \inf \left\{ t > 0 : \int _\Xi \exp \left( \left|\frac{f}{t} \right|^{1/a} \right) \, \textrm{d}\mu \leqslant 1 \right\} < \infty . \end{aligned}$$

The norm (1.3) is equivalent to the dual norm on $\exp \textrm{L}^{1/a}$ so that there is *C* depending only on $(\Xi ,\mu )$ such that for all $f \in \textrm{L}(\log \textrm{L})^a(\Xi )$ and $u \in \exp \textrm{L}^{1/a}(\Xi )$ the Hölder-type inequality

1.4 $$\begin{aligned} \left\| fu \right\| _{\textrm{L}^1(\Xi )} \leqslant C \left\| f \right\| _{\textrm{L}(\log \textrm{L})^a(\Xi )} \left\| u \right\| _{\exp \textrm{L}^{1/a}(\Xi )} \end{aligned}$$

holds.

Given a conformal class $\mathcal {C}$ and $\Omega \in \mathcal {C}$ with boundary conformal factor $\beta$ we consider the weighted Steklov problem

1.5 $$\begin{aligned} {\left\{ \begin{array}{ll} \Delta u = 0 &{} \text {in } \Omega _\mathcal {C}; \\ \partial _\nu u = \beta \sigma u &{} \text {on } \partial \Omega _\mathcal {C}, \end{array}\right. } \end{aligned}$$

The spectrum of this weighted problem is discrete and accumulates at infinity if the trace operator $\textrm{W}^{1,2}(\Omega _\mathcal {C}) \rightarrow \textrm{L}^2(\partial \Omega _\mathcal {C}, \beta \, \textrm{d}\ell _{g_\mathcal {C}})$ is compact, see [13, Sections 3 and 4]. This is the case if $\beta \in \textrm{L}\log \textrm{L}(\partial \Omega _\mathcal {C})$, see Proposition 2.2.

#### Theorem 1.7

Let $\mathcal {C}$ be a conformal class and $(\Omega ,g) \in \mathcal {C}$ be a surface with boundary conformal regularity $\textrm{L}\log \textrm{L}$. Then, problems (1.1) and (1.5) are *isospectral* in the sense that $\sigma _k(\Omega ) = \sigma _k(\Omega _\mathcal {C},\beta )$ for all $k \in {\mathbb {N}}$.

#### Remark 1.8

A simple computation shows that Theorem 1.7 holds when the boundary is smooth, see for example [22, Lemma 3.3]. If the boundary is sufficiently rough, isospectrality is not a priori clear, and is resolved through the study of composition operators between Sobolev spaces with appropriately chosen norm. In a similar way, the existence of those bounded composition operators gives rise to isospectrality of the weighted Neumann problems, see [19]. This issue was not previously addressed in the literature on the Steklov problem on Lipschitz domains, cf. [16, Proposition 2.1.4].

Our main technical theorem is

#### Theorem 1.9

Let $\Omega$ be a surface with boundary of boundary conformal regularity $\textrm{L}\log \textrm{L}$. Then its Steklov eigenvalues satisfy the asymptotic formula (1.2).

Equivalently, for any surface $\Omega$ with smooth boundary and $\beta \in \textrm{L}\log \textrm{L}(\partial \Omega )$, the eigenvalues of the weighted problem (1.5) satisfy the asymptotic formula (1.2), with $\text {Vol}_{d-1}(\partial \Omega )$ replaced by $\int _{\partial \Omega } \beta \, \textrm{d}\ell _g$.

The equivalence of the two formulations follows from Theorem 1.7. Note that in view of Remark 1.5, Theorems 1.7 and 1.9 imply Theorem 1.1.

### Variational Characterisation and Natural Domains for the Steklov Problem

On a surface with boundary $\Omega$, consider the Sobolev space

$$\begin{aligned} \textrm{W}^{1,2}(\Omega ) := \left\{ f \in \textrm{L}^2(\Omega ) : \left|\nabla f \right| \in \textrm{L}^2(\Omega ) \right\} , \end{aligned}$$

where $\nabla f$ is the weak gradient. If $\Omega$ is a surface with Lipschitz boundary, there are two equivalent norms on $\textrm{W}^{1,2}(\Omega )$:

1.6 $$\begin{aligned} \left\| f \right\| ^2_{\textrm{W}^{1,2}(\Omega )} = \int _\Omega \left|\nabla f \right|^2 \, \textrm{d}v_g + \int _\Omega f^2 \, \textrm{d}v_g, \end{aligned}$$

and

1.7 $$\begin{aligned} \left\| f \right\| ^2_{\textrm{W}^{1,2}_\partial (\Omega )} = \int _\Omega \left|\nabla f \right|^2 \, \textrm{d}v_g + \int _{\partial \Omega } f^2 \, \textrm{d}\ell _g. \end{aligned}$$

The norm (1.6) is the standard one and is commonly used in interior problems, for instance the Neumann problem. On the other hand the norm (1.7) is a natural norm of choice for the Steklov problem. When the boundary is only some collection of Jordan curves these norms may not be equivalent, even when the boundary has finite perimeter (one can show that this is the case for domains with fast cusps as defined in Sect. 4.2). By the Meyers–Serrin theorem, for any surface with boundary $\Omega$ the space $\textrm{W}^{1,2}(\Omega )$ is the completion of

$$\begin{aligned} \mathcal {W}(\Omega ) := \left\{ f \in \textrm{C}^\infty (\Omega ) : \left\| f \right\| _{\textrm{W}^{1,2}(\Omega )} < \infty \right\} \end{aligned}$$

under the $\left\| \cdot \right\| _{\textrm{W}^{1,2}(\Omega )}$ norm, which motivates the following definition:

#### Definition 1.10

Let $\Omega$ be a surface with boundary. The boundary Sobolev space $\textrm{W}^{1,2}_\partial (\Omega )$ is defined as the completion of

$$\begin{aligned} \mathcal {W}_\partial (\Omega ) := \left\{ f : \overline{\Omega }\rightarrow {\mathbb {R}}: f \in \textrm{C}^\infty (\Omega ) \text { and } \left\| f \right\| _{\textrm{W}^{1,2}_\partial (\Omega )} < \infty \right\} \end{aligned}$$

under the $\left\| \cdot \right\| _{\textrm{W}^{1,2}_\partial (\Omega )}$ norm.

Again, for surfaces with sufficiently regular boundary, $\textrm{W}^{1,2}(\Omega )$ and $\textrm{W}^{1,2}_\partial (\Omega )$ are isomorphic. We give the following condition for their equivalence in terms of interior and boundary conformal regularity.

#### Proposition 1.11

Let $\Omega$ be a surface with boundary with both interior *and* boundary conformal regularity $\textrm{L}\log \textrm{L}$. Then, $\textrm{W}^{1,2}(\Omega )$ and $\textrm{W}_\partial ^{1,2}(\Omega )$ are isomorphic.

The appropriate space to define the Steklov problem (especially when $\textrm{W}^{1,2}(\Omega )$ and $\textrm{W}^{1,2}_\partial (\Omega )$ are not isomorphic) is $\textrm{W}^{1,2}_\partial (\Omega )$, see [26]. The Steklov eigenvalues $\sigma _k(\Omega )$ satisfy the variational characterisation

$$\begin{aligned} \sigma _k(\Omega ) = \inf _{E_k} \sup _{u \in E_k \setminus \left\{ 0 \right\} } \frac{\int _\Omega \left|\nabla u \right|^2 \, \textrm{d}v_g}{\int _{\partial \Omega } u^2 \, \textrm{d}\ell _g}, \end{aligned}$$

where $E_k$ is a $k+1$ dimensional subspace of $\textrm{W}_\partial ^{1,2}(\Omega )$. For the weighted problem on $\Omega _\mathcal {C}$, we have that for $\beta \in \textrm{L}\log \textrm{L}(\Omega _\mathcal {C})$ the weighted Steklov eigenvalues satisfy the characterisation

$$\begin{aligned} \sigma _k(\Omega _\mathcal {C},\beta ) = \inf _{E_k} \sup _{u \in E_k \setminus \left\{ 0 \right\} } \frac{\int _{\Omega _\mathcal {C}} \left|\nabla u \right|^2 \, \textrm{d}v_g}{\int _{\partial \Omega _\mathcal {C}} u^2 \beta \, \textrm{d}\ell _g}, \end{aligned}$$

where again $E_k$ is a $k+1$ dimensional subspace of $\textrm{W}_\partial ^{1,2}(\Omega _\mathcal {C})$. The isospectrality Theorem 1.7 is a consequence of the following result on composition operator between Sobolev spaces:

#### Proposition 1.12

Let $\mathcal {C}$ be a conformal class and $(\Omega ,g) \in \mathcal {C}$ be a surface with boundary. Let $\varphi : \Omega _\mathcal {C}\rightarrow \Omega$ be a conformal diffeomorphism with boundary conformal factor $\beta \in \textrm{L}\log \textrm{L}$. Then, the composition operator

$$\begin{aligned} \varphi ^* : \textrm{W}^{1,2}_\partial (\Omega ) \rightarrow \textrm{W}^{1,2}_\partial (\Omega _\mathcal {C}) \qquad \varphi ^* f := f \circ \varphi \end{aligned}$$

induced by $\varphi$ is an isomorphism.

### Plan of the Paper

The text of this paper is organised as follows: Sect. 2 is concerned with the proof of Theorem 1.7. We then use the variational isospectrality to prove that domains of boundary conformal regularity $\textrm{L}\log \textrm{L}$ have discrete Steklov spectrum. Section 3 is dedicated to proving Theorem 1.9, expanding on the theory of spectral asymptotics for variational eigenvalues developed in [9, 42]. In Sect. 4 we give a few examples of domains satisfying the hypotheses of Theorem 1.9. Finally, in Sect. 5 we discuss some further extensions and applications of our methods, in particular to the Steklov problem with an indefinite weight and to the Neumann eigenvalue problem.

## Isospectrality and Composition Operators

We first prove the following lemma about composition operators on some Orlicz spaces, in a fashion similar to [19, Theorem 4], which is stated for Lebesgue spaces:

### Lemma 2.1

For $j \in \left\{ 1,2 \right\}$, let $(\Xi _j,\mu _j)$ be measure spaces with finite measure, $\varphi : \Xi _1 \rightarrow \Xi _2$ be measurable and suppose that the pushforward measure $\varphi _*(\mu _1) = \beta \mu _2$, where $\beta : \Xi _2 \rightarrow (0,\infty )$. Then, $\varphi$ induces a bounded composition operator

$$\begin{aligned} \varphi ^* : \exp \textrm{L}^2(\Xi _2) \rightarrow \textrm{L}^2(\Xi _1), \qquad \varphi ^* f := f \circ \varphi \end{aligned}$$

if and only if $\beta \in \textrm{L}\log \textrm{L}(\Xi _2)$.

### Proof

To prove that the condition $\beta \in \textrm{L}\log \textrm{L}(\Xi _2)$ is sufficient, assume that $f \in \exp \textrm{L}^2(\Xi _2)$, so that $\left|f \right|^2 \in \exp \textrm{L}(\Xi _2)$. Since $\textrm{L}\log \textrm{L}(\Xi _2)$ is a reflexive space with dual $\exp \textrm{L}(\Xi _2)$, we can compute

2.1 $$\begin{aligned} \begin{aligned} \int _{\Xi _1} \left|\varphi ^* f \right|^2 \, \textrm{d}\mu _1&= \int _{\Xi _2} \left|f \right|^2 \beta \, \textrm{d}\mu _2 \\&\leqslant \left\| \beta \right\| _{\textrm{L}\log \textrm{L}(\Xi _2)} \left\| f^2 \right\| _{\exp \textrm{L}(\Xi _2)}\\&= \left\| \beta \right\| _{\textrm{L}\log \textrm{L}(\Xi _2)} \left\| f \right\| _{\exp \textrm{L}^2(\Xi _2)}^2. \end{aligned} \end{aligned}$$

Let us now show that the condition is necessary. Indeed, if $\beta \not \in \textrm{L}\log \textrm{L}(\Xi _2)$, it is not a bounded linear functional on $\exp \textrm{L}(\Xi _2)$, so that we can make the second integral in the first line of (2.1) arbitrarily large with an appropriate choice of $\left|f \right|^2 \in \exp \textrm{L}(\Xi _2)$. $\square$

In the next proposition, we show compactness of a weighted boundary trace operator. The proof is similar in nature to the ideas in [13, Example 3.19 (iii)] where the weight is instead in the interior.

### Proposition 2.2

Let $\Omega$ be a surface with smooth boundary and $0 \leqslant \beta \in \textrm{L}\log \textrm{L}(\partial \Omega )$, $\beta \not \equiv 0$. Then, the trace operator $T_\beta : \textrm{W}^{1,2}(\Omega ) \rightarrow \textrm{L}^2(\partial \Omega , \beta \textrm{d}\ell )$ is compact.

### Proof

Define $\theta : \partial \Omega \rightarrow {\mathbb {R}}$ as $\theta := \frac{1}{\beta }\varvec{1}_{\left\{ \beta > 0 \right\} }$. Consider the diagramwhere *T* is the trace operator and $M_h$ is the operator of multiplication by the function *h*. The trace operator *T* is bounded; $\exp \textrm{L}^2(\partial \Omega )$ is in fact the optimal target space on $\partial \Omega$ for bounded traces from $\textrm{W}^{1,2}(\Omega )$, see [10, Example 5.3]. By Hölder’s inequality (1.4) between $\exp L(\partial \Omega )$ and $\textrm{L}\log \textrm{L}(\partial \Omega )$ there exists, $C > 0$ such that

$$\begin{aligned} \left\| M_{\sqrt{\beta }} f \right\| _{\textrm{L}^2(\partial \Omega )}^2= & {} \int _{\partial \Omega } f^2 \beta \, \textrm{d}\ell \leqslant C\left\| f^2 \right\| _{\exp \textrm{L}(\partial \Omega )} \left\| \beta \right\| _{\textrm{L}\log \textrm{L}(\partial \Omega )}\\= & {} C\left\| f \right\| _{\exp \textrm{L}^2(\partial \Omega )}^2 \left\| \beta \right\| _{\textrm{L}\log \textrm{L}(\partial \Omega )}. \end{aligned}$$

In other words, $M_{\sqrt{\beta }}$ is bounded with norm at most $C \left\| \beta \right\| _{\textrm{L}\log \textrm{L}(\partial \Omega )}$. As for $M_{\sqrt{\theta }}$, we have that

$$\begin{aligned} \left\| M_{\sqrt{\theta }}f \right\| ^2_{\textrm{L}^2(\partial \Omega ;\beta \, \textrm{d}\ell )} = \int _{\partial \Omega \cap \left\{ \beta > 0 \right\} } f^2 \, \textrm{d}\ell \leqslant \left\| f \right\| _{\textrm{L}^2(\partial \Omega )}^2 \end{aligned}$$

Thus, for a probably different constant $C>0$ independent of $\beta$ we have

$$\begin{aligned} \left\| T_\beta \right\| \leqslant C \left\| \beta \right\| _{\textrm{L}\log \textrm{L}(\partial \Omega _\mathcal {C})}. \end{aligned}$$

To prove compactness, it is sufficient to prove that $M_{\sqrt{\beta }} \circ T$ is compact. If $\beta$ is a nonnegative smooth function, the composition can instead be factored asand compactness therefore follows from the usual trace restriction theorem $\textrm{W}^{1,2}(\Omega ) \rightarrow \textrm{L}^2(\partial \Omega )$, see [29, Theorem 2.6.2]. By density of smooth functions in $\textrm{L}\log \textrm{L}$, for every $\varepsilon > 0$, there is a nonnegative $\beta _\varepsilon \in \textrm{C}^\infty (\partial \Omega )$ such that $\left\| \beta - \beta _\varepsilon \right\| _{\textrm{L}\log \textrm{L}(\partial \Omega )} < \varepsilon$ and $\beta _\varepsilon \leqslant \beta$ almost everywhere, so that $\sqrt{\beta } - \sqrt{\beta _\varepsilon } \leqslant \sqrt{\beta - \beta _\varepsilon }$. but then

$$\begin{aligned} \left\| M_{\sqrt{\beta }} \circ T - M_{\sqrt{\beta _\varepsilon }} \circ T \right\| \leqslant \left\| M_{\sqrt{\beta - \beta _\varepsilon }} \right\| \left\| T \right\| \leqslant \left\| \beta - \beta _\varepsilon \right\| _{\textrm{L}\log \textrm{L}(\partial \Omega )} \left\| T \right\| \leqslant \varepsilon \left\| T \right\| . \end{aligned}$$

Thus $M_{\sqrt{\beta }} \circ T$ is a norm limit of compact operators hence compact itself and $T_\beta$ is compact also. $\quad \square$

We now have the right tools to prove the composition Propositon 1.12, following the structure of the proof of [19, Theorem 6]:

### Proof of Proposition 1.12

Let $f \in \mathcal {W}_\partial (\Omega )$ as in Definition 1.10. Invariance of the Dirichlet energy under conformal diffeomorphisms tells us that

$$\begin{aligned} \left\| \nabla f \right\| _{\textrm{L}^2( \Omega )} = \left\| \nabla (\varphi ^* f) \right\| _{\textrm{L}^2(\Omega _\mathcal {C})}. \end{aligned}$$

By Lemma 2.1, since the boundary conformal factor is in $\textrm{L}\log \textrm{L}$, $\varphi$ induces the bounded composition operator $(\varphi ^{-1})^*: \exp \textrm{L}^2(\partial \Omega _\mathcal {C}) \rightarrow \textrm{L}^2(\partial \Omega )$. Therefore, for every $a \in {\mathbb {R}}$, we have that

2.2 $$\begin{aligned} \begin{aligned} \left|a \right|&= {{\,\textrm{Per}\,}}(\Omega )^{-1/2} \left\| a \right\| _{\textrm{L}^2(\partial \Omega )} \\ {}&\leqslant {{\,\textrm{Per}\,}}(\Omega )^{-1/2} \left( \left\| f \right\| _{\textrm{L}^2(\partial \Omega )} + \left\| f- a \right\| _{\textrm{L}^2(\partial \Omega )} \right) \\ {}&\leqslant {{\,\textrm{Per}\,}}(\Omega )^{-1/2} \left( \left\| f \right\| _{\textrm{L}^2(\partial \Omega )} + \left\| (\varphi ^{-1})' \right\| _{\textrm{L}\log \textrm{L}(\partial \Omega _\mathcal {C})} \left\| \varphi ^* f- a \right\| _{\exp \textrm{L}^2(\partial \Omega _\mathcal {C})} \right) . \end{aligned} \end{aligned}$$

We claim that there exists a constant $C > 0$ such that

$$\begin{aligned} \begin{aligned} \left\| \varphi ^* f \right\| _{\textrm{L}^2(\partial \Omega _\mathcal {C})}&\leqslant \inf _{a \in {\mathbb {R}}} \left( \left\| a \right\| _{\textrm{L}^2(\partial \Omega _\mathcal {C})} + \left\| \varphi ^*f - a \right\| _{\textrm{L}^2(\partial \Omega _\mathcal {C})}\right) \\&\leqslant \inf _{a \in {\mathbb {R}}} \left( {{\,\textrm{Per}\,}}(\Omega )^{-1/2} {{\,\textrm{Per}\,}}(\Omega _\mathcal {C})^{1/2} + C\left\| 1 \right\| _{\textrm{L}\log \textrm{L}(\partial \Omega _\mathcal {C})}\right) \\&\qquad \left( \left\| f \right\| _{\textrm{L}^2(\partial \Omega )} + \left\| \varphi ^*f - a \right\| _{\exp \textrm{L}^2(\partial \Omega _\mathcal {C})} \right) . \end{aligned} \end{aligned}$$

Indeed, the first inequality is just the triangle inequality. We then use (2.2) to estimate the first term, and inequality (1.4) together with the relations $\left\| h \right\| _{\textrm{L}^2}^2=\left\| h^2 \right\| _{\textrm{L}^1}$, $\left\| h \right\| _{\exp \textrm{L}^2}^2=\left\| h^2 \right\| _{\exp \textrm{L}^1}$ to estimate the second.

The space $\exp \textrm{L}^2(\partial \Omega _\mathcal {C})$ is the optimal target space for trace operators from $\textrm{W}^{1,2}(\Omega _\mathcal {C})$, and this is equivalent to the validity of a Poincaré trace inequality, see [10, Theorems 1.3 and 5.3],

$$\begin{aligned} \inf _a \left\| \varphi ^* f - a \right\| _{\exp \textrm{L}^2(\partial \Omega _\mathcal {C})} \leqslant C \left\| \nabla \varphi ^* f \right\| _{\textrm{L}^2(\Omega _\mathcal {C})}. \end{aligned}$$

Combining the previous display formulas gives us the existence of some constant $C > 0$ such that

$$\begin{aligned} \left\| \varphi ^*f \right\| _{\textrm{W}_\partial ^{1,2}(\Omega _\mathcal {C})} \leqslant C \left\| f \right\| _{\textrm{W}_\partial ^{1,2}(\Omega )}. \end{aligned}$$

Since $\mathcal {W}_\partial (\Omega )$ is dense in $\textrm{W}^{1,2}_\partial (\Omega )$, the pullback $\varphi ^*$ extends to the whole space as a bounded operator as well. Proving the analogous result for $(\varphi ^{-1})^*$ is simpler. Since $\Omega _\mathcal {C}$ has smooth boundary, the spaces $\textrm{W}^{1,2}(\Omega _\mathcal {C})$ and $\textrm{W}^{1,2}_\partial (\Omega _\mathcal {C})$ are isomorphic. Compactness (in fact, boundedness is enough here) of the trace operator $\textrm{W}^{1,2}(\Omega _\mathcal {C}) \rightarrow \textrm{L}^2(\partial \Omega _\mathcal {C}, \beta \, \textrm{d}\ell _{g_\mathcal {C}})$ obtained in Proposition 2.2 then implies that for every $h \in \mathcal {W}(\Omega _\mathcal {C})$,

$$\begin{aligned} \left\| (\varphi ^{-1})^* h \right\| _{\textrm{W}_\partial ^{1,2}(\Omega )}^2 =\left\| \nabla h \right\| _{\textrm{L}^2(\Omega _\mathcal {C})}^2 + \left\| h \right\| _{\textrm{L}^2(\partial \Omega _\mathcal {C}, \beta \textrm{d}\ell _{g_\mathcal {C}})}^2 \leqslant C \left\| h \right\| _{\textrm{W}_\partial ^{1,2}(\Omega _\mathcal {C})}^2. \end{aligned}$$

By density we once again have that $(\varphi ^{-1})^*$ extends to the whole space as a bounded operator, completing the proof. $\quad \square$

We can now prove Theorem 1.7.

### Proof of Theorem 1.7

Let $E_k$ be a $k+1$ dimensional subspace of $\textrm{W}_\partial ^{1,2}(\Omega )$. Then, by Proposition 1.12, $\varphi ^*(E_k)$ is a $k+1$ dimensional subspace of $\textrm{W}_\partial ^{1,2}(\Omega _\mathcal {C})$, and for every $u \in E_k$,

$$\begin{aligned} \frac{\int _\Omega \left|\nabla u \right|^2 \, \textrm{d}v_g}{\int _{\partial \Omega } u^2 \, \textrm{d}\ell _g}= \frac{\int _{\Omega _\mathcal {C}} \left|\nabla u \right|^2 \, \textrm{d}v_{g_\mathcal {C}}}{\int _{\partial \Omega _\mathcal {C}} u^2 \beta \, \textrm{d}\ell _{g_\mathcal {C}}}. \end{aligned}$$

This implies directly that $\sigma _k(\Omega _\mathcal {C},\beta ) \leqslant \sigma _k(\Omega )$. The analogous reasoning with $(\varphi ^{-1})^*$ instead of $\varphi ^*$ gives the reverse inequality. $\quad \square$

In order to prove Proposition 1.11, we extend the results of [19] to a slightly more singular interior conformal factor.

### Lemma 2.3

Let $\mathcal {C}$ be a conformal class and $(\Omega ,g) \in \mathcal {C}$ be a surface with boundary, which has interior conformal regularity $\textrm{L}\log \textrm{L}$ through the conformal diffeomorphism $\varphi : \Omega _\mathcal {C}\rightarrow \Omega$. Then, the composition operator

$$\begin{aligned} \varphi ^* : \textrm{W}^{1,2}(\Omega ) \rightarrow \textrm{W}^{1,2}(\Omega _\mathcal {C}) \qquad \varphi ^* f := f \circ \varphi \end{aligned}$$

induced by $\varphi$ is an isomorphism.

### Proof

The proof is essentially identical to the proof of Proposition 1.12. We replace the result on the trace operators $\textrm{W}_\partial ^{1,2}(\Omega _\mathcal {C}) \rightarrow \exp \textrm{L}^2(\partial \Omega _\mathcal {C})$ and the corresponding Poincaré inequality with the optimal Sobolev embedding $\textrm{W}^{1,2}(\Omega _\mathcal {C}) \rightarrow \exp \textrm{L}^2(\Omega _\mathcal {C})$, and use the fact that the interior conformal factor $\left|\textrm{d}\varphi \right|^2 \in \textrm{L}\log \textrm{L}$ to get a bounded composition operator $\exp \textrm{L}^2(\Omega _\mathcal {C}) \rightarrow \textrm{L}^2(\Omega )$. $\quad \square$

We can now prove Proposition 1.11.

### Proof (Proof of Proposition 1.11)

Since $\Omega _\mathcal {C}$ is a surface with smooth boundary, the spaces $\textrm{W}^{1,2}(\Omega _\mathcal {C})$ and $\textrm{W}_\partial ^{1,2}(\Omega _\mathcal {C})$ are isomorphic, via some linear map $\iota$. Interior conformal regularity $\textrm{L}\log \textrm{L}$ provides us with an isomorphism $\varphi ^*: \textrm{W}^{1,2}(\Omega ) \rightarrow \textrm{W}^{1,2}(\Omega _\mathcal {C})$ and boundary conformal regularity $\textrm{L}\log \textrm{L}$ with an isomorphism $\varphi _\partial ^*: \textrm{W}_\partial (\Omega ) \rightarrow \textrm{W}^{1,2}(\Omega _\mathcal {C})$. The composition $(\varphi _\partial ^*)^{-1} \circ \iota \circ \varphi ^*$ provides the desired isomorphism. $\quad \square$

## Spectral Asymptotics

### Eigenvalue Counting Functions of Compact Operators

We first present some known results about spectral asymptotics of compact operators defined via quadratic forms. These results can be found in the works of Suslina [42], Birman–Solomyak [9], and Sukochev–Zanin [40], in a more general form. For the convenience of the reader we state them here in a form which is specific for our purposes.

Let $\mathcal {H}$ be a Hilbert space and $K \in \mathcal {K}(\mathcal {H})$ be a self-adjoint nonnegative compact operator. The non-zero spectrum of *K* consists of a discrete set of nonincreasing nonnegative eigenvalues $\left\{ \lambda _j(K): j \in {\mathbb {N}} \right\}$ counted with multiplicity and converging to 0. The variational characterisation of the eigenvalues yields

3.1 $$\begin{aligned} \lambda _j(K) = \max _{E_j \subset \mathcal {H}} \min _{u \in E_j \setminus \left\{ 0 \right\} } \frac{(Ku,u)}{(u,u)}, \end{aligned}$$

where $E_j$ ranges over *j* dimensional subspaces. Note that the operator *K* can be equivalently defined via the associated bilinear form appearing in the numerator (3.1); we will use this observation further on. For $\lambda >0$, define the eigenvalue counting function

$$\begin{aligned} n(\lambda ;K) := \# \left\{ j : \lambda _j(K) > \lambda \right\} , \end{aligned}$$

and, for a given $\alpha > 0$, the functionals

$$\begin{aligned} \overline{n}_\alpha (K) = \limsup _{\lambda \searrow 0} \lambda ^\alpha n(\lambda ;K) \text { and } \underline{n}_\alpha (K) = \liminf _{\lambda \searrow 0} \lambda ^\alpha n(\lambda ;K). \end{aligned}$$

Note that if $\overline{n}_\alpha (K) = \underline{n}_\alpha (K) = C_\alpha$, then

$$\begin{aligned} n(\lambda ;K) = C_\alpha \lambda ^{-\alpha }(1 + o_{}\left( 1 \right) ). \end{aligned}$$

We make use of the following general properties of this counting function, which are collected in [9, Appendix 1], see also the references therein:

#### Lemma 3.1

The following properties hold:

1. [9, Lemma 1.16] For any $\alpha > 0$, the functionals $\overline{n}_\alpha (K)$ and $\underline{n}_\alpha (K)$ are invariant under compact perturbations of the inner product on $\mathcal {H}$, as well as restriction to subspaces of finite codimension.
2. [9, Lemma 1.18 and its proof], [42, Lemma 1.5], Weyl–Fan Ky lemma. Let $K_1 \leqslant K_2 \in \mathcal {K}(\mathcal {H})$ be nonnegative self-adjoint compact operators. Then, for any $\alpha >0$,
  $$\begin{aligned} \left|\overline{n}_\alpha (K_1)^{\frac{1}{1 + \alpha }} - \overline{n}_\alpha (K_2)^{\frac{1}{1 + \alpha }} \right| \leqslant \overline{n}_\alpha (K_2 - K_1)^{\frac{1}{1+\alpha }} \end{aligned}$$
  and
  $$\begin{aligned} \left|\underline{n}_\alpha (K_1)^{\frac{1}{1 + \alpha }} - \underline{n}_\alpha (K_2)^{\frac{1}{1 + \alpha }} \right| \leqslant \overline{n}_\alpha (K_2 - K_1)^{\frac{1}{1+\alpha }}. \end{aligned}$$
3. [9, Lemma 1.15] Let $K_1 \in \mathcal {K}(\mathcal {H}_1)$ and $K_2 \in \mathcal {K}(\mathcal {H}_2)$ be nonnegative self-adjoint compact operators. Let $B: \mathcal {H}_1 \rightarrow \mathcal {H}_2$ be a bounded operator such that $(K_1 u,u)_{\mathcal {H}_1} = 0$ for all $u \in \ker B$. If there is $a > 0$ such that for all $u \in \mathcal {H}_1\setminus \ker B$
  $$\begin{aligned} \frac{(K_1 u,u)_{\mathcal {H}_1}}{(u,u)_{\mathcal {H}_1}} \leqslant a \frac{(K_2 B u,B u)_{\mathcal {H}_2}}{(Bu,Bu)_{\mathcal {H}_2}}, \end{aligned}$$
  then for all $\lambda > 0$, $n(\lambda ;K_1) \leqslant n(a^{-1}\lambda ;K_2)$ for all $\lambda > 0$.

We will use these abstract results in the concrete situation where $\mathcal {H}= \textrm{W}^{1/2,2}(\Gamma )$, where $\Gamma$ is a finite collection of smooth curves with length measure $\textrm{d}\ell$. For $\beta : \Gamma \rightarrow [0,\infty )$ let $K_\beta$ be the operator in $\textrm{W}^{1/2,2}(\Gamma )$ be defined by the bilinear form

3.2 $$\begin{aligned} (K_{\beta } u, v)_{\mathcal {H}} = \int _\Gamma u \overline{v} \beta \, \textrm{d}\ell , \quad u,v \in {\text {Dom}}(K_{\beta }). \end{aligned}$$

The following lemma essentially goes back to the work of Solomyak [39], see also [38]. It is a direct reinterpretation of [40, Lemma 4.4] (cf. [35, Theorem 2.1]) in view of the variational characterisation of the eigenvalue counting function [9, Lemma 1.14].

#### Lemma 3.2

Let $\Gamma$ be a finite collection of smooth curves and $0 \leqslant \beta \in \textrm{L}\log \textrm{L}(\Gamma )$. Let $K_{\beta }$ be the self-adjoint operator on $\textrm{W}^{1/2,2}(\Gamma )$ defined via the bilinear form (3.2). Then there exists a constant $C(\Gamma )>0$ such that

$$\begin{aligned} n(\lambda ;K_{\beta }) \leqslant C(\Gamma ) \lambda ^{-1} \left\| \beta \right\| _{\textrm{L}\log \textrm{L}(\Gamma )}. \end{aligned}$$

We now have the required tools to prove Theorem 1.9.

### Proof of Theorem 1.9

We turn to the second, equivalent, statement. Recall that the eigenvalues of the weighted Steklov problem on a surface with smooth boundary are characterised variationally as

3.3 $$\begin{aligned} \sigma _k(\Omega ,\beta ) = \min _{E_k} \max _{U \in E_k \setminus \left\{ 0 \right\} } \frac{\int _{\Omega } \left|\nabla U \right|^2 \, \textrm{d}A_{g}}{\int _{\partial \Omega } u^2 \beta \, \textrm{d}\ell _{g}}. \end{aligned}$$

Here $E_k \subset \textrm{W}^{1,2}(\Omega )$ (which is isomorphic to $\textrm{W}^{1,2}_\partial (\Omega )$) is a $k+1$ dimensional subspace, and $u:=\tau U$, where $\tau : \textrm{W}^{1,2}(\Omega ) \rightarrow \textrm{W}^{1/2,2}(\partial \Omega )$ is the trace operator, which is continuous. Here and further on we adopt the following convention: capital letters denote functions in the interior, and the corresponding lower case letters denote their boundary traces.

Let

$$\begin{aligned} \mathcal {X}:= \left\{ V \in \textrm{W}^{1,2}(\Omega ) : \int _{\partial \Omega } v \beta \, \textrm{d}\ell _{g} = 0 \right\} . \end{aligned}$$

be the orthogonal complement in $\textrm{L}^2(\partial \Omega ;\beta \, \textrm{d}\ell _{g})$ to the kernel of the weighted Dirichlet-to-Neumann map, that is to the constant functions. We equip $\mathcal {X}$ with the inner product

$$\begin{aligned} (U,U)_\mathcal {X}= \int _{\Omega } \left|\nabla U \right|^2 \, \textrm{d}A_{g}. \end{aligned}$$

Let us define an operator $Q_\beta$ on $\mathcal {X}$ via the bilinear form

3.4 $$\begin{aligned} (Q_\beta U, V)_{\mathcal {X}} = \int _{\partial \Omega } u \, v \, \beta \, \textrm{d}\ell _g, \quad u,v \in \mathcal {X}. \end{aligned}$$

Clearly, we have that

3.5 $$\begin{aligned} \lambda _k(Q_\beta ) = \max _{E_k \subset \mathcal {X}} \min _{u \in E_k \setminus \left\{ 0 \right\} } \frac{\int _{\partial \Omega } u^2 \beta \, \textrm{d}\ell _{g}}{(U,U)_\mathcal {X}}. \end{aligned}$$

In view of (3.5) and (3.3) we have that for $k \geqslant 1$, $\sigma _k(\Omega ,\beta )^{-1} = \lambda _k(Q_\beta )$, so that

3.6 $$\begin{aligned} N(\sigma ;M,\beta ) - 1 = n(\sigma ^{-1};Q_\beta ), \end{aligned}$$

where we have subtracted one on the left to account for the eigenvalue zero. Let us find the asymptotics of $n(\sigma ^{-1};Q_\beta )$ as $\sigma ^{-1} =:\lambda \searrow 0$. It follows from Lemma 3.1(1) that the asymptotics of $n(\lambda ;Q_\beta )$ does not change if we first replace $(U,U)_\mathcal {X}$ with $(U,U)_\mathcal {X}+ (U,U)_{\textrm{L}^2(\Omega )}$ (this is a compact perturbation), and then lift the orthogonality condition, in order to consider $U \in \textrm{W}^{1,2}(\Omega )$ as in (3.3). By the density of smooth functions in $\textrm{L}\log \textrm{L}$, for every $\varepsilon > 0$ we can find a smooth $\beta _\varepsilon \in \textrm{C}^\infty (\partial \Omega )$ such that $\left\| \beta - \beta _\varepsilon \right\| _{\textrm{L}\log \textrm{L}} < \varepsilon$. Without loss of generality, we suppose that $\beta _\varepsilon \leqslant \beta$ almost everywhere so that $Q_\beta - Q_{\beta _\varepsilon }$ is a positive operator. Since we know, by the general theory of pseudodifferential operators, that as $\lambda \searrow 0$

$$\begin{aligned} n(\lambda ;Q_{\beta _\varepsilon }) = \frac{\lambda ^{-1}}{\pi } \int _{\partial \Omega } \beta _\varepsilon \, \textrm{d}\ell _g + o_{}\left( \lambda ^{-1} \right) , \end{aligned}$$

it is sufficient by Lemma 3.1(2) to show that

$$\begin{aligned} n(\lambda ;Q_\beta - Q_{\beta _\varepsilon }) \leqslant C \lambda ^{-1} \left\| \beta - \beta _\varepsilon \right\| _{\textrm{L}\log \textrm{L}}, \end{aligned}$$

with *C* depending only on $\Omega$. It immediately follows from (3.4) that $\ker \tau \subset \ker (Q_\beta - Q_{\beta _\varepsilon })$. Defining $K_{\beta }$ as in Lemma 3.2 with $\Gamma = \partial \Omega$, we have that for all $U \in \textrm{W}^{1,2}(\Omega )$,

$$\begin{aligned} ( (K_{\beta } - K_{\beta _\varepsilon }) u,u)_{\textrm{W}^{1/2,2}(\partial \Omega )} = ((Q_\beta - Q_{\beta _\varepsilon }) U, U)_{\textrm{W}^{1,2}(\Omega )} \end{aligned}$$

By the trace theorem, we also have that there exists $C_\Omega$ such that

$$\begin{aligned} (\tau U,\tau U)_{\textrm{W}^{1/2,2}(\partial \Omega )} \leqslant C_\Omega (U,U)_{\textrm{W}^{1,2}(\Omega )}. \end{aligned}$$

By applying first Lemma 3.1(3) then Lemma 3.2 we deduce that

$$\begin{aligned} \begin{aligned} n(\lambda ;Q_\beta - Q_{\beta _\varepsilon })&\leqslant n(C_\Omega \lambda ;K_{\beta } - K_{\beta _\varepsilon }) \\&\leqslant C_\Omega ' \lambda ^{-1} \left\| \beta -\beta _\varepsilon \right\| _{\textrm{L}\log \textrm{L}(\partial \Omega )} \\&\leqslant C_\Omega ' \lambda ^{-1} \varepsilon . \end{aligned} \end{aligned}$$

Since this holds for arbitrary $\varepsilon > 0$, we deduce that

$$\begin{aligned} n(\lambda ;Q_\beta ) = \frac{\lambda ^{-1}}{\pi } \int _{\partial \Omega } \beta \, \textrm{d}\ell _g + o_{}\left( \lambda ^{-1} \right) \end{aligned}$$

and in view of (3.6) this completes the proof of the theorem.

## Examples

In this last section, we present examples of domains having conformal regularity $\textrm{L}\log \textrm{L}$ and explore the sharpness of Theorem 1.9. We give planar domains as example, but they extend in a straightforward manner to domains in a complete Riemannian surface.

### Chord-Arc Domains

Recall that a Jordan domain $\Omega \subset \mathbb {R}^2$ is called a *chord-arc* (or *Lavrentiev*) *domain* if there exists a constant *C* such that for any $x,y \in \partial \Omega$

$$\begin{aligned} {{\,\textrm{dist}\,}}_{\partial \Omega } (x,y) \leqslant C {{\,\textrm{dist}\,}}_{{\mathbb {R}}^2}(x,y), \end{aligned}$$

where the left-hand sides denotes the length of the shortest arc of the boundary joining *x* and *y*, and the right-hand side denotes the distance between *x* and *y* in $\mathbb {R}^2$. It is clear that any Lipschitz domain is a chord-arc domain. The class of chord-arc domains is larger than Lipschitz and includes, in particular, the domain bounded between two logarithmic spirals. Note that domains with cusps are not chord-arc.

There is a large literature on the conformal regularity of chord-arc domains, see, for instance [21] and references therein. The next result is well-known. We outline its proof below for the convenience of the reader.

#### Proposition 4.1

Let $\Omega \subset \mathbb {R}^2$ be a chord-arc domain and let $\varphi :\mathbb {D}\rightarrow \Omega$ be a conformal map. Then $\varphi ' \in \textrm{L}^p(\partial \Omega )$ for some $p>1$.

#### Proof

Since $\Omega$ is a chord-arc domain, by [45, Theorem 1] we have that $\varphi ' \in \textrm{A}_q$ for some $q>1$, where $\textrm{A}_q$ denotes a Muckenhoupt class of weights (see, for instance [12, Section VI.6] for a definition). By [12, Corollary 6.10], every Muckenhoupt weight on $\partial \Omega$ of class $\textrm{A}_q$, $q > 1$ is in $\textrm{L}^p(\partial \Omega )$ for some $p > 1$, which is our claim.$\square$

### Domains with Cusps

Let $\Omega \subset \mathbb {R}^2$ be a domain with boundary $\partial \Omega$ which is a finite union of smooth curves. If two curves meet at an interior angle zero we say that they form an *outward cusp*, and if the interior angle is equal to $2\pi$ we say that they form an *inward cusp*.

Let $x_0$ to be the tip of a cusp, $\varphi : \Omega _\mathcal {C}\rightarrow \Omega$ be a conformal diffeomorphism, and set $\Omega _\mathcal {C}\ni z_0 = \varphi ^{-1}(x_0)$. If $x_0$ is the tip of an inward cusp, then $\varphi '(z_0)=0$. In fact domains with inward cusps have $\varphi ' \in \textrm{C}^{0,1}(\partial \Omega )$ [30, Theorem 3.9]. A typical example is the standard cardioid domain defined in polar coordinates as $\left\{ (r,\theta ): r=2(1+\cos \theta ) \right\}$, for which $\varphi (z)=(z+1)^2$.

Consider now domains with outward cusps. Suppose that in a neighbourhood of the outward cusp at $x_0$ the boundary of $\Omega$ consists of two smooth curves $\gamma _1(t)$, $\gamma _2(t)$, where *t* is the arc length parameter and $\gamma _1(0) = \gamma _2(0) = x_0$. We say that $\Omega$ has a *slow* cusp at $x_0$ if there is $\alpha \in (0,1)$ (the speed of the cusp) such that

$$\begin{aligned} \lim _{t \searrow 0}\frac{\left|\gamma _1(t) - \gamma _2(t) \right|}{t^{1 + \alpha }} = s_\alpha > 0 \end{aligned}$$

In turn if there is *C* such that $t^{-2} \left|\gamma _1(t) - \gamma _2(t) \right| \leqslant C < \infty$ for all $t > 0$ we say that $\Omega$ has a *fast* cusp at $x_0$.

It is shown in [26] that whenever a domain has a fast cusp, the Dirichlet-to-Neumann map does not have a compact resolvent. Therefore, its spectrum is not discrete and Weyl’s law can not hold. However, the Dirichlet-to-Neumann map for a domain whose boundary is Lipschitz except at a finite number of slow cusps has a compact resolvent, and hence a discrete spectrum.

Suppose now that $x_0$ is the tip of a cusp of speed $\alpha$. Applying [23, Proposition 2.10] to [32, Corollary 1], we see that as $z \rightarrow z_0$ the conformal factor $|\textrm{d}\varphi (z)|$ behaves asymptotically as

4.1 $$\begin{aligned} \left| \textrm{d}\varphi (z) \right| = O\left( |z - z_0|^{-1} \left( -\log (\left|z - z_0 \right|\right) ^{-1-\frac{1}{\alpha }}\right) . \end{aligned}$$

A direct calculation gives that $\left|\textrm{d}\varphi \right| \in \textrm{L}\log \textrm{L}$ if and only if $0< \alpha < 1$. In other words, precisely for those $\alpha$ for which the spectrum is discrete. This shows that Theorem 1.9 gives in a sense an optimal condition for the validity of Weyl’s law.

Let us summarize the results of this subsection in the following:

#### Proposition 4.2

Let $\Omega \subset \mathbb {R}^2$ be a domain with piecewise smooth boundary, possibly with interior and exterior cusps. If all exterior cusps are slow then Weyl’s law (1.2) holds for the counting function of the Steklov eigenvalues on $\Omega$. Moreover, if $\Omega$ has at least one fast cusp, then the Steklov spectrum of $\Omega$ is not discrete.

#### Remark 4.3

It would be interesting to understand if there exist domains for which the Steklov spectrum is discrete but the Weyl’s law (1.2) does not hold. To construct such an example one needs to find a domain $\Omega$ with the boundary conformal factor in $\textrm{L}^1 \setminus \textrm{L}\log \textrm{L}$, and yet for which the resolvent of the Dirichlet-to-Neumann map is still compact. We note that in terms of the weighted problem it seems like this would require going beyond the Orlicz scale: indeed, for every $0 \leqslant a < 1$ one can find $\beta \in \textrm{L}(\log \textrm{L})^a(\partial \Omega )$ so that the embedding $\textrm{W}^{1,2}(\partial \Omega ) \rightarrow \textrm{L}^2(\partial \Omega , \beta \textrm{d}\ell )$ is not compact, following the proof found in [13, Example 3.19].

## Further Remarks and Extensions

### The Steklov Problem with Indefinite Weight

Suppose for now that $\Omega$ is a surface with smooth boundary, and given $\beta : \partial \Omega \rightarrow {\mathbb {R}}$ consider the Steklov problem with an indefinite weight:

5.1 $$\begin{aligned} {\left\{ \begin{array}{ll} \Delta u = 0 &{} \text {in } \Omega ; \\ \partial _\nu u = \beta \sigma u &{} \text {on } \partial \Omega . \end{array}\right. } \end{aligned}$$

Indefinite eigenvalue problems of this type have been considered in the literature, see for example [1, 36, 41, 42]. If $0 \not \equiv \beta \in \textrm{L}\log \textrm{L}(\partial \Omega )$ is such that $\left\{ \beta > 0 \right\}$ and $\left\{ \beta < 0 \right\}$ both have positive measure in $\partial \Omega$, then the non-zero eigenvalues form two sequences $\left\{ \sigma _k^\pm (\Omega ,\beta ): k \in {\mathbb {N}} \right\}$ consisting of the positive and negative eigenvalues, accumulating respectively at $\pm \infty$. To define the variational principle, let us first denote

$$\begin{aligned} \left\| f \right\| _{\textrm{W}_\partial ^{1,2}(\Omega ;\beta )}^2 = \int _\Omega \left|\nabla f \right|^2 \, \textrm{d}v_g + \int _{\partial \Omega } f^2 \left|\beta \right|\, \textrm{d}\ell _g \end{aligned}$$

and

$$\begin{aligned} \mathcal {W}_\partial (\Omega ;\beta ) := \left\{ f : \overline{\Omega }\rightarrow {\mathbb {R}}: f \in \textrm{C}^\infty (\Omega ) \text { and } \left\| f \right\| _{\textrm{W}_\partial ^{1,2}(\Omega ;\beta )} < \infty \right\} . \end{aligned}$$

We denote by $\textrm{W}^{1,2}_\partial (\Omega ;\beta )$ the closure of $\mathcal {W}_\partial (\Omega ;\beta )$ under the $\left\| \cdot \right\| _{\textrm{W}^{1,2}_\partial (\Omega ;\beta )}$ norm, and $\mathcal {X}$ to be the subset of $\textrm{W}^{1,2}_\partial (\Omega ;\beta )$ orthogonal to $\beta$. Following [9, 42], the non-zero eigenvalues of problem (5.1) satisfy the variational principle

$$\begin{aligned} \frac{\pm 1}{\sigma ^\pm _k(\Omega ,\beta )} = \min _{F_k} \max _{u \in F_k \setminus \left\{ 0 \right\} } \pm \frac{\int _{\partial \Omega } u^2 \beta \, \textrm{d}\ell _g}{\int _{\Omega } \left|\nabla u \right|^2 \, \textrm{d}A_g}, \end{aligned}$$

where $F_k$ is a codimension $k-1$ subspace of $\mathcal {X}$. Denoting by

$$\begin{aligned} N^\pm (\sigma ;\Omega ,\beta ) := \#\left\{ k : 0< \pm \sigma _k^\pm (\Omega ,\beta )< \sigma \right\} \end{aligned}$$

the counting functions for each of those sequences, it follows from the work of Birman–Solomyak [7, 8] (see [31, Theorem 6.1] for a modern proof, in English) that if $\beta$ is smooth, then

5.2 $$\begin{aligned} N^\pm (\sigma ;\Omega ,\beta ) = \frac{\sigma }{\pi } \int _{\partial \Omega } \beta _\pm \, \textrm{d}\ell + o_{}\left( \sigma \right) \end{aligned}$$

where $\beta _\pm = \max \left\{ 0,\pm \beta \right\}$ are the positive and negative parts of $\beta$. This formula is valid whether or not $\beta$ takes both positive and negative values. Using the same methods as in Sect. 3 allows us to extend this result to $\beta \in \textrm{L}\log \textrm{L}(\partial \Omega )$. We note that the results in Lemma 3.1 are in fact proven in [9, 42] for operators with both positive and negative spectrum, with the obvious redefinition of the functions $\overline{n}_\alpha ^\pm$ and $\underline{n}_\alpha ^\pm$.

When $\Omega$ has non-smooth boundary, we consider once again a conformal map $\varphi : \Omega _\mathcal {C}\rightarrow \Omega$. If the product $\varphi ^* \beta \left|\textrm{d}\varphi \right| \in \textrm{L}\log \textrm{L}(\partial \Omega _\mathcal {C})$, then the proof of Proposition 1.11 carries through and $\varphi$ induces an isomorphism $\varphi ^*$ between $\textrm{W}^{1,2}_\partial (\Omega ;\beta )$ and $\textrm{W}^{1,2}_\partial (\Omega _\mathcal {C};\varphi ^* \beta )$. If both $\varphi ^* \beta$ and $\varphi ^*\beta \left|\textrm{d}\varphi \right|$ are in $\textrm{L}\log \textrm{L}(\partial \Omega _\mathcal {C})$ then

$$\begin{aligned} \textrm{W}_\partial ^{1,2}(\Omega _\mathcal {C};\varphi ^*\beta ) \cong \textrm{W}_\partial ^{1,2}(\Omega _\mathcal {C}) \cong \textrm{W}_\partial ^{1,2}(\Omega _\mathcal {C};\varphi ^*\beta \left|\textrm{d}\varphi \right|) \end{aligned}$$

and as in Theorem 1.7 problem (5.1) is isospectral to

$$\begin{aligned} {\left\{ \begin{array}{ll} \Delta u = 0 &{} \text {in } \Omega _\mathcal {C}\\ \partial _\nu u = \left|\textrm{d}\varphi \right| \varphi ^* \beta \sigma u &{} \text {on } \partial \Omega _\mathcal {C}. \end{array}\right. } \end{aligned}$$

We see directly that a sufficient condition for having the Weyl law (5.2) is also that $\left|\textrm{d}\varphi \right| \varphi ^* \beta \in \textrm{L}\log \textrm{L}(\partial \Omega _\mathcal {C})$. For any surface with Lipschitz boundary, we have that $\left|\textrm{d}\varphi \right|$ and $\left|\textrm{d}\varphi \right|^{-1}$ are in $\textrm{L}^p$ for some $p > 1$ with Hölder conjugate $p'$, see [4, proof of Lemma 5.1]. Therefore, if $\varphi ^* \beta \in \textrm{L}^q(\partial \Omega _\mathcal {C})$ for $q > p'$, the Weyl law (5.2) holds. Arguing as in [44, Theorem 4], see also [19, Theorem 4], one can show that for $q > p'$ the map $\varphi$ induces a bounded composition operator $\varphi ^*: \textrm{L}^{\frac{qp}{p-1}}(\partial \Omega ) \rightarrow \textrm{L}^q(\partial \Omega _\mathcal {C})$. Therefore, a sufficient condition for the Weyl law to hold is $\beta \in \textrm{L}^r(\partial \Omega )$, for some $r > p^2/(p-1)^2$. In particular, if $\Omega$ is a Lipschitz domain and $\beta$ is in some Orlicz space contained in $\textrm{L}^q(\partial \Omega )$ for any $q < \infty$, then the weighted Steklov problem, definite or not, satisfies the Weyl law (5.2).

### The Neumann Problem

The methods developed in this paper can be also applied to the Neumann problem

$$\begin{aligned} {\left\{ \begin{array}{ll} - \Delta _g u = \lambda u &{} \text {in } \Omega \\ \partial _\nu u = 0 &{} \text {on } \partial \Omega . \end{array}\right. } \end{aligned}$$

In this case, the conformal map $\varphi : \Omega _\mathcal {C}\rightarrow \Omega$ gives rise to the variationally isospectral weighted Neumann problem

$$\begin{aligned} {\left\{ \begin{array}{ll} - \Delta _g u = \lambda \left|\textrm{d}\varphi \right|^2 u &{} \text {in } \Omega _\mathcal {C}\\ \partial _\nu u = 0 &{} \text {on } \partial \Omega _\mathcal {C}. \end{array}\right. } \end{aligned}$$

If the boundary is regular enough, the Neumann spectrum is discrete and we aim for a Weyl law of the form

5.3 $$\begin{aligned} N_{\text {Neu}}(\lambda ) = \frac{{\text {Area}}(\Omega )}{4\pi } \lambda + o_{}\left( \lambda \right) . \end{aligned}$$

This problem is well studied, and, in particular, we already know that the Weyl law holds for a large class of domains with rough boundary. For instance, it is shown in [28] that (5.3) for every domain whose boundary is of the Hölder class $\textrm{C}^{0,\alpha }$ for $\alpha > 1/2$, see also [27] It is also shown in [28] that domains with finite straight cusps of any speed satisfy (5.3). Moreover, sharp remainder estimates have been obtained in many cases.

A straightforward adaptation of the methods developed in this paper yields an alternative proof of (5.3) provided $\left|\textrm{d}\varphi \right|^2 \in \textrm{L}\log \textrm{L}(\Omega _\mathcal {C})$. This is shown in essentially the same way as Lemma 3.2. While this approach does not give sharp remainder estimates, it is significantly more elementary. Using (4.1), we note that the class of domains for which $\left|\textrm{d}\varphi \right|^2 \in \textrm{L}\log \textrm{L}(\Omega _\mathcal {C})$ includes any cusp of polynomial speed.

## Data Availability

Data sharing not applicable to this article as no datasets were generated or analysed in this current study.
